# Age-related differences in functional brain network segregation are consistent with a cascade of cerebrovascular, structural, and cognitive effects

**DOI:** 10.1162/netn_a_00110

**Published:** 2020-02-01

**Authors:** Tania S. Kong, Caterina Gratton, Kathy A. Low, Chin Hong Tan, Antonio M. Chiarelli, Mark A. Fletcher, Benjamin Zimmerman, Edward L. Maclin, Bradley P. Sutton, Gabriele Gratton, Monica Fabiani

**Affiliations:** Beckman Institute, University of Illinois at Urbana-Champaign, IL, USA; Psychology Department, University of Illinois at Urbana-Champaign, IL, USA; Department of Psychology, Northwestern University, IL, USA; Department of Neurology, Northwestern University, IL, USA; Beckman Institute, University of Illinois at Urbana-Champaign, IL, USA; Beckman Institute, University of Illinois at Urbana-Champaign, IL, USA; Division of Psychology, Nanyang Technological University, Singapore; Department of Pharmacology, National University of Singapore, Singapore; Beckman Institute, University of Illinois at Urbana-Champaign, IL, USA; Department of Neuroscience, Imaging and Clinical Sciences, University G. D’Annunzio of Chieti-Pescara, Chieti, Italy; Beckman Institute, University of Illinois at Urbana-Champaign, IL, USA; Beckman Institute, University of Illinois at Urbana-Champaign, IL, USA; Beckman Institute, University of Illinois at Urbana-Champaign, IL, USA; Beckman Institute, University of Illinois at Urbana-Champaign, IL, USA; Department of Bioengineering, University of Illinois at Urbana-Champaign, IL, USA; Beckman Institute, University of Illinois at Urbana-Champaign, IL, USA; Psychology Department, University of Illinois at Urbana-Champaign, IL, USA; Beckman Institute, University of Illinois at Urbana-Champaign, IL, USA; Psychology Department, University of Illinois at Urbana-Champaign, IL, USA

**Keywords:** Aging, Resting-state functional connectivity (rsFC), Cerebrovascular health, Optical brain arterial pulse (pulse-DOT), White matter signal abnormalities (WMSAs), Cortical thickness

## Abstract

Age-related declines in cognition are associated with widespread structural and functional brain changes, including changes in resting-state functional connectivity and gray and white matter status. Recently we have shown that the elasticity of cerebral arteries also explains some of the variance in cognitive and brain health in aging. Here, we investigated how network segregation, cerebral arterial elasticity (measured with pulse-DOT—the arterial pulse based on diffuse optical tomography) and gray and white matter status jointly account for age-related differences in cognitive performance. We hypothesized that at least some of the variance in brain and cognitive aging is linked to reduced cerebrovascular elasticity, leading to increased cortical atrophy and white matter abnormalities, which, in turn, are linked to reduced network segregation and decreases in cognitive performance. Pairwise comparisons between these variables are consistent with an exploratory hierarchical model linking them, especially when focusing on association network segregation (compared with segregation in sensorimotor networks). These findings suggest that preventing or slowing age-related changes in one or more of these factors may induce a neurophysiological cascade beneficial for preserving cognition in aging.

## Author Summary

Age-related declines in cognition are associated with widespread structural and functional brain changes as well as changes in the elasticity of cerebral arteries. In this study, using an exploratory hierarchical model as a guide, and novel measures of cerebral arterial elasticity (pulse-DOT—the arterial pulse based on diffuse optical tomography), we show, for the first time, that cerebral arterial stiffness is strongly correlated with measures of functional brain network segregation, even after partialing out the effects of age. These findings suggest that preventing cerebral arterial stiffening could induce a neurophysiological cascade beneficial for preserving cognition in aging.

## INTRODUCTION

Aging, even in the apparent absence of disease, is often accompanied by cognitive decline, albeit with large individual differences (Fabiani, 2012; Salthouse, 2012; Shaw, Schultz, Sperling, & Hedden, 2015). These age-related decreases in cognitive functioning have been linked to functional and structural brain changes, including alterations in resting-state functional connectivity (rsFC; see Ferreira & Busatto, 2013, for a review), cortical atrophy (Salat et al., 2004; Thambisetty et al., 2010), white matter health (DeCarli et al., 1995; Sullivan & Pfefferbaum, 2006; Yang, Tsai, Liu, Huang, & Lin, 2016), and cerebral arterial elasticity (Chiarelli et al., 2017; Fabiani et al., 2014; Tan et al., 2017, 2016). Resting-state FC is an important tool for gaining insight into the intrinsic organization of the brain. It is now widely understood that, even in the absence of any explicit tasks or goals, brain regions spontaneously group into intrinsic connectivity networks with temporally correlated activity (Power, Barnes, Snyder, Schlaggar, & Petersen, 2012; Power, Schlaggar, & Petersen, 2014; Yeo et al., 2011). Aging is known to be accompanied by widespread changes in rsFC (see Ferreira & Busatto, 2013 for a review), manifesting as decreased rsFC within brain networks and increased rsFC across networks. These changes may be quantified as decreased network modularity or system segregation (Betzel et al., 2014; Chan, Park, Savalia, Petersen, & Wig, 2014; Geerligs, Renken, Saliasi, Maurits, & Lorist, 2015). They appear to be particularly prominent in the default mode network (DMN; Damoiseaux et al., 2008) and in networks related to higher level cognitive functions (as compared with networks involved in sensory and motor processing; e.g., Andrews-Hanna et al., 2007; Chan et al., 2014).

There is also evidence that age-related differences in rsFC are linked to age-related differences in cognition (Andrews-Hanna et al., 2007; Chan et al., 2014; Geerligs et al., 2015). For example, using seed analysis, Andrews-Hanna et al. (2007) demonstrated that the rsFC between DMN seeds in the medial-prefrontal cortex and in the posterior-cingulate was positively correlated with performance in a composite memory measure. Similarly, using graph theory analyses, Chan et al. (2014) demonstrated that the segregation of association system networks was predictive of memory function, with greater system segregation associated with higher memory function. This was not the case for the segregation of networks related to sensorimotor processing. Other studies have also reported similar relationships between rsFC and cognition by using other network measures such as modularity and local efficiency, although these results do not always survive correction for multiple comparisons (e.g., Geerligs et al., 2015).

Finally, it has been reported that network connectivity measures may also be predictive of cognitive improvements as a result of training in older adults. For example, older adults with higher baseline measures of network segregation (or modularity) showed greater cognitive gains as a result of cognitive training (Gallen et al., 2016) and aerobic-based training (Baniqued et al., 2018). Thus, converging evidence suggests that rsFC not only changes systematically with aging but also plays an important role in age-related changes in cognition.

Although this evidence points to the importance of rsFC for understanding age-related differences in brain function, other factors are also known to contribute to individual differences in age-related cognitive decline. For instance, cortical thinning occurs during aging (Salat et al., 2004; Thambisetty et al., 2010) and has been shown to correlate with individual differences in cognitive performance. In a longitudinal study collecting MRI data over 8 years, participants who had the greatest amount of cortical thinning at baseline (compared with participants having less evidence of thinning) later exhibited clinical levels of impairments in cognition (Pacheco, Goh, Kraut, Ferrucci, & Resnick, 2015). Similarly, in an extensive series of studies conducted by Dickerson and colleagues, cortical thinning has also been shown to be predictive of Alzheimer’s disease onset and progression (Bakkour, Morris, & Dickerson, 2009; Dickerson et al., 2011; Racine, Brickhouse, Wolk, & Dickerson, 2018).

White matter integrity also generally decreases with age (Fletcher et al., 2016; Gordon et al., 2008; Raz, Ghisletta, Rodrigue, Kennedy, & Lindenberger, 2010; Sullivan & Pfefferbaum, 2006; Yang et al., 2016) and is correlated with individual differences in cognition in normally aging adults (G. Gratton, Wee, Rykhlevskaia, Leaver, & Fabiani, 2008; Jolly et al., 2017, 2016; Sullivan & Pfefferbaum, 2006; Yang et al., 2016). There is, in fact, evidence that age-related declines in white matter integrity obtained with MR diffusivity methods may be related to age-related reductions in rsFC. For example, it has been shown that white matter integrity was positively correlated with within-DMN rsFC even after controlling for age (Andrews-Hanna et al., 2007). However, another study examining the relationship between FC in seven networks and white matter MR diffusivity measures including mean anisotropy, fractional anisotropy, and tract length did not find any relationship between FC and structural connectivity (Tsang et al., 2017).

In light of these contradictory results, it is possible to hypothesize that white matter lesions, manifesting as white matter signal abnormalities (WMSAs; e.g., DeCarli et al., 1995; Tan et al., in press) may be a more sensitive measure for examining the relationship between white matter integrity and age-related rsFC in older adults. WMSAs are based on the identification of spots of hypointense (on T1 images) or hyperintense (in T2 images) MR signal and are thought to reflect isolated areas of demyelination. Recent studies show that the presence of WMSAs is closely related to amyloid deposition measures and is indicative of preclinical Alzheimer’s disease in healthy older adults (Kandel et al., 2016; Lindemer, Greve, Fischl, Augustinack, & Salat, 2017). WMSAs are also inversely related to measures of fluid intelligence (Leaper et al., 2001). These findings, coupled with the temporal match between increasing WMSAs and conversion to mild cognitive impairment in older adults (DeCarli et al., 1995), suggest that WMSAs may be a stronger indicator of advanced stages of cognitive decline than other measures of white matter integrity (Maniega et al., 2015).

White matter lesions such as WMSAs are thought to be caused primarily by vascular dysfunctions (Bots et al., 1993; Longstreth et al., 1996; Moroni et al., 2018), largely related to arteriosclerosis (i.e., stiffening of the arteries). Cortical thinning may also be related to arterial issues, although the mechanisms for this relationship are less well established (e.g., Marshall, Asllani, Pavol, Cheung, & Lazar, 2017). Good cardiovascular and cardiorespiratory health are also known to promote and maintain cognitive performance in aging (e.g., Colcombe et al., 2004; Crichton, Elias, Davey, & Alkerwi, 2014; Gordon et al., 2008). However, studies of the impact of vascular health on cognition thus far have most often been based on peripheral indices of arterial elasticity (e.g., by measuring the carotid-femoral pulse delay; see Maillard et al., 2016; Badji et al., 2019). To more directly assess *cerebrovascular* health, we have recently developed measures of the cerebral arterial pulse based on diffuse optical tomography (pulse-DOT; Chiarelli et al., 2017; Fabiani et al., 2014; Tan et al., 2017, 2016). This approach allows for the assessment and mapping of arterial elasticity in the brain, both globally and regionally. In the current study, we use this novel pulse-DOT approach to assess arterial elasticity *within the cerebral vascular tree*, therefore allowing for the investigation of more direct associations between cerebrovascular status and brain measures such as cortical thinning, WMSAs, and rsFC parameters. One of the indices of arterial elasticity afforded by pulse-DOT is a measure of the shape of the pulse wave during the interval between a peak systole and the subsequent peak diastole (which we refer to as pulse relaxation function, or PReFx). PReFx describes the way in which arteries return to their original size, after dilating to accommodate the oxygenated blood bolus generated by a heart pulsation. This reflects the relative overlap of the forward wave generated by the systole and the backward wave generated by the peripheral resistance (i.e., arterioles). This overlap (or lack thereof) is largely due to the speed of propagation of the pulse wave between the measurement point and the point of peripheral resistance, which, in turn, is determined by the rigidity (or lack of elasticity, i.e., arterial stiffness or arteriosclerosis) of the arterial wall (Oliver & Webb, 2003): The greater the rigidity, the greater the pulse-wave velocity, and the greater the temporal overlap between the forward and backward waves, as indicated by the convexity of the pulse-wave shape (i.e., PReFx) during the systole-diastole interval (Chiarelli et al., 2017; Fabiani et al., 2014).

Our previous work has shown that cerebral arterial elasticity measured with pulse-DOT is not only associated with age and cardiorespiratory fitness, but also with behavioral measures of cognitive flexibility (Fabiani et al., 2014; Tan et al., 2017). Furthermore, these studies have also shown correlations between pulse-DOT measures and white and gray matter volumes, not only across individuals but also across different brain regions within the same individuals (Chiarelli et al., 2017). As WMSAs have been linked to vascular factors such as hypertension (e.g., Longstreth et al., 1996; Moroni et al., 2018), it can be hypothesized that a reduction in arterial elasticity (i.e., arterial stiffness or arteriosclerosis) is an important contributor to the development of WMSAs (Tan et al., in press) and cortical thinning in aging.

Here we hypothesized that at least some of the effects of aging on the structural integrity of the brain, its functional network segregation, and cognitive performance may be due to the effects of arteriosclerosis. As such we tested pairwise correlations between cerebral arterial elasticity, as measured by PReFx, rsFC segregation, cortical thinning, WMSAs, and cognitive performance. Specifically, we hypothesized that individual differences in network segregation would be correlated with differences in PReFx, with lower arterial elasticity (indexed by a smaller PReFx) being associated with reduced segregation. Although this relationship may be driven in part by age, as older adults are likely to exhibit both lower arterial elasticity and lower network segregation, we predicted that this relationship would also explain residual variance when age was controlled for. The mechanisms linking PReFx and rsFC segregation are likely to be complex and multiply determined. Individual differences in arteriosclerosis, caused by long-term lifestyle factors such as diet, exercise, and stress (Bowie, Clements, Gratton, & Fabiani, in press), and exacerbated by aging, are known to attack brain structure, and especially the white matter, which is essential to efficient connectivity. In turn, this is likely to result in lower cognitive performance, especially in domains that are more vulnerable to aging (e.g., episodic memory and reasoning, with the latter being a key component of fluid intelligence; Lindenberger & Baltes, 1997). Maintaining a healthy and elastic cerebral arterial system, therefore, is likely to preserve brain and cognitive function, including the maintenance of network segregation, which is typical of younger adults and correlated with higher cognitive performance. In the final part of this paper we introduce, and provide an initial test for, an exploratory hierarchical model that links these structural, functional, and cognitive factors together.

## Materials and Methods

### Participants

Forty-nine healthy right-handed (as assessed by the Edinburgh Handedness Inventory; Oldfield, 1971) adults, evenly distributed by age and gender (approximately 8 people per decade, 50% female; total sample: 23 males, mean age: 47.29; age range: 18–75 years) were recruited from the Urbana-Champaign community. These were the same participants included in the Chiarelli et al. (2017) and Tan et al. (2017, in press) publications focused on arterial elasticity data. The current study includes, for the first time, functional connectivity analyses based on a resting-state fMRI protocol, which have not been previously analyzed or published. Participants reported no history of neurological or psychiatric disorders and had no signs of dementia (as assessed by a score > 51 on the modified Mini-Mental Status Examination [mMMSE]; Mayeux, Stern, Rosen, & Leventhal, 1981) or depression (as assessed by the Beck’s Depression Inventory; Beck, Steer, & Brown, 1996). Informed consent was provided by each participant. All procedures were approved by the Institutional Review Board of the University of Illinois.

Data from two participants were excluded because of excess movement during the resting-state fMRI data acquisition (i.e., participants with fewer than 50 frames remaining for at least one of the two runs were excluded; see section *Functional connectivity* and Supporting Information S1.1 for specific details). Data from one additional participant were excluded because of technical issues during optical data acquisition. All analyses and results described below are based on a final sample of 46 participants (21 males, mean age: 46.41 [*SD* = 17.32]; age range: 18–75).

### Data Acquisition

Data for each participant were collected during a cognitive testing session, an optical imaging session, and a session during which both structural and functional MRI data were acquired.

##### Cognitive testing session.

In this session the following neuropsychological tests were administered: Logical Memory I and II tasks from the Wechsler Memory Scale–Fourth Edition (WMS-IV; Wechsler, 2009) to measure episodic memory; the Trail Making Tests A and B (Corrigan & Hinkeldey, 1987), to measure processing speed and working memory; the Controlled Oral Word Association subtest of the Multilingual Aphasia Examination (a measure of verbal fluency using the letters CFL; Benton & Hamsher, 1989); the OSPAN task (Unsworth, Heitz, Schrock, & Engle, 2005) to assess working memory capacity under load; Raven’s progressive matrices (Raven, Raven, & Court, 2003) and the Kaufmann Brief Intelligence Test Second Edition (K-BIT2; Kaufman & Kaufman, 2004) to assess, respectively, cognitive flexibility and IQ; the vocabulary subtest of the Shipley Institute of Living Scale (Shipley, 1940).

##### Structural and functional MRI session.

Participants underwent a scanning session in a 3-Tesla Siemens Trio MR scanner, using a 12-channel head coil. Resting-state images were acquired during an echo planar imaging sequence with the following pulse parameters: repetition time (TR) 2,000 ms; echo time (TE) = 25 ms; 38 contiguous interleaved 3-mm slices; flip angle (FA) = 90°; voxel sizes = 2.6 × 2.6 × 3.0 mm. Two 6-minute scans were collected during rest while participants fixated on a cross in the center of the screen.

A 3D T1-weighted anatomical scan for each participant was also acquired using an MPRAGE sequence with the following pulse parameters: TR = 1,900 ms; TE = 2.32 ms; 192 sagittal slices; slice thickness = 0.90 mm; FA = 9°; voxel sizes = 0.9 ×0.9 × 0.9 mm, field-of-view (FOV) = 172.8 × 230 × 230 mm. The FOV for both MPRAGE and resting-state scans covered the entire head.

##### Optical imaging session.

Arterial elasticity (pulse-DOT) data were obtained during a resting-state optical imaging session, in which seated participants fixated on a cross in the center of a screen. Optical data were acquired with a multichannel frequency-domain oximeter (ISS Imagent, Champaign, Illinois) equipped with 128 laser diodes (64 emitting light at 690 nm and 64 at 830 nm) and 24 photomultiplier tubes. Time-division multiplexing was employed so that each detector picked up light from 16 different sources at different times within a multiplexing cycle at a sampling rate of 39.0625 Hz. The light was transmitted to the scalp by using single-optic fibers (0.4-mm core) and from the scalp back to the photomultiplier tubes by using fiber bundles (3-mm diameter). The fibers were held in place using soft, but semirigid, custom-built helmets, fitted to participants based their head circumference.

During this session, after the helmet was set up, the locations of the optodes were marked digitally to improve spatial accuracy during later data processing. Fiducial markers were placed on each participant’s left and right preauricular points and on the nasion. These fiducial points, optode locations, and other scalp locations were digitized with a Polhemus FastTrak 3D digitizer (accuracy: 0.8 mm; Colchester, VT) by using a recording stylus and three head-mounted receivers, which allowed for small movements of the head in between measurements. Optode locations and structural MRI data were then coregistered using the fiducials and a surface-fitting Levenberg and Marquardt algorithm (Chiarelli, Maclin, Fabiani, & Gratton, 2015).

The pulse-DOT measurements were based on a high-density, large FOV optode montage, covering the majority of the cortical mantle. Two 6-minute resting-state blocks were recorded for each of four different optical recording montages, which were aggregated during analysis for maximum cortical coverage. The helmet was never removed across the entire optical session to remain faithful to the digitized locations of the optical sensors. A total of 384 channels (192 at 830 nm and 192 at 690 nm) were acquired for each montage, with source-detector distances varying between 15 and 80 mm, for a total of 1,536 channels covering most of the scalp surface. The FOV for the pulse-DOT measures is related to the montage used (which, in this case, covered the entire scalp) and to the penetration of diffuse optical imaging (approximately 30 mm from the head’s surface, encompassing most of the outer cortex).

Concurrently with pulse-DOT data acquisition (but not during the MR scanning) we also recorded the electrocardiogram (EKG), using a Brain-Vision recorder and a Brain-Vision professional BrainAmp integrated amplifier system (Brain Products, Germany). This concurrent and time-locked acquisition allowed us to synchronize the optical pulse data to the R wave of the EKG and ensure that the same pulse was examined regardless of location. Specifically, lead 1 of the EKG (left wrist referenced to right wrist) was recorded with a sampling rate of 200 Hz and a band-pass filter of 0.1–100 Hz. The exact timing of each R-wave peak was determined by searching for peak points exceeding a voltage threshold (scaled for each participant) and dismissing any peak points outside the normal range of interbeat intervals. The identification of each peak was verified by visual inspection, and false detections were eliminated.

### Data Processing

##### Cognitive testing data.

We adapted the methods described in the Supplementary Results section of Chan et al. (2014) and categorized our cognitive tests into the same a priori constructs (Chan et al., 2014; see Table 1). In line with Chan et al. (2014), construct scores were calculated by averaging the z-scores across the different cognitive tests that made up a construct. Since age effects are of interest in our study, we performed this calculation on raw scores instead of age-adjusted scores, even for tests that normally adjust their final scores for age (i.e., KBIT-2 and CFL). Furthermore, since the Trail Making test A used for the processing speed construct gives *higher* scores (i.e., longer times) for *slower* participants, we inverted its z-scores to make it comparable to the results of Chan et al. (2014), since they used the digit comparison and WAIS digit symbol tests where participants with lower performance had lower scores. We did not have tests that were similar to those used for the Mental Control construct in Chan et al. (2014; CANTAB Stop Signal Task and ETS Card Rotation) so we did not include this construct.

**Table 1.** A priori cognitive constructs

A priori cognitive construct	Associated cognitive test(s)
Episodic memory	WMS-IV Logical memory (immediate and delayed)
	WMS-IV Verbal pairing (immediate and delayed)
Processing speed	Trail Making task A
Verbal fluency	CFLˆ
Working memory	Trail Making test A minus Trail Making test B
	OSPAN
Reasoning	Raven’s progressive matrices
	KBIT-2 non-verbal scoresˆ
Verbal ability	Shipley vocabulary subtest
	KBIT-2 verbal scoresˆ

*Note.* We composed a priori constructs based on the methods and construct categories of Chan et al. (2014). Scores for the tests associated with each construct were converted to *z*-scores and averaged with other tests associated with the same construct. Tests whose scores are normally adjusted for age are indicated withˆ. Since we are interested in age effects, we performed the *z*-score conversions on the raw scores instead of the age-adjusted scores. WMS = Wechsler Memory Scale; CFL = verbal fluency test (letters CFL); KBIT = Kaufman Brief Intelligence Test.

##### White matter signal abnormalities and cortical thickness.

Cortical reconstruction and volumetric segmentation were performed on the structural MPRAGE images by using the FreeSurfer 5.3 image analysis suite (http://surfer.nmr.mgh.harvard.edu/; Fischl & Dale, 2000). This same automated procedure also yielded estimates of total intracranial volume (Buckner et al., 2004). Cortical thickness estimates provided by FreeSurfer were obtained for each of the 50 regions of the Desikan-Killiany atlas that are superficial and can be investigated using diffuse optical imaging methods (to make these analyses consistent with the Pulse-DOT analyses, see below). The average cortical thickness across all these regions was used as an estimate of overall cortical thickness for each individual. White matter signal abnormalities (WMSAs), which appear as hypointense on T1-weighted images, were labeled automatically using FreeSurfer 5.3’s probabilistic procedure (Fischl et al., 2002). The results of this automatic segmentation were inspected for errors and corrected where needed (https://surfer.nmr.mgh.harvard.edu/fswiki/FsTutorial/TroubleshootingData). The WMSA variable was log-transformed because of its positively skewed distribution and adjusted for intracranial volume and sex.

##### Pulse-DOT measures of arterial stiffness.

Arterial elasticity was quantified using PReFx (Chiarelli et al., 2017; Fabiani et al., 2014). As mentioned in the Introduction, PReFx describes the temporal overlap between the forward and backward waves generated during each cardiac cycle. A greater overlap between these two waves is associated with low PReFx values and indexes arteriosclerosis, whereas a small overlap is associated with high PReFx values and higher arterial elasticity. Also, as a reminder, PReFx refers to arterial elasticity in the arterial tract connecting the point of measurement (which in optical recordings is close to the surface of the cortex) with the place where peripheral resistance occurs (and therefore the backward wave is generated), that is, downstream relative to the point where the measurements are taken. Therefore, PReFx, although measured superficially, is not only sensitive to arterial stiffening occurring in superficial regions but also to stiffening occurring in deeper regions, such as those where WMSAs are most likely to occur. This is different from other pulse parameters (such as pulse amplitude or transit time) that are instead more sensitive to arterial elasticity at the point of measurement (amplitude) and at points upstream from the point of measurement (transit time). This is the reason we focus on the PReFx parameter in the current paper.

To derive the PReFx measures, consistent with Chiarelli et al. (2017), the optical DC intensity data (i.e., the average measures of the amount of light produced by a specific source and reaching a specific detector during a multiplexed 1.6-ms interval) at 830 nm were normalized, movement corrected (Chiarelli et al., 2015), and band-pass filtered between 0.5 and 5 Hz by using a Butterworth filter. The arterial pulse waveform for each channel was obtained by averaging the DC light intensity time locked to the peak of the R wave of the EKG, ensuring that the same pulse cycle was measured at all locations. Three-dimensional reconstruction of the pulse waveform across the head was estimated using a finite element method (FEM) applied to the diffusion equation (Ishimaru, 1989; Paulsen & Jiang, 1995) for the forward model, and an inverse procedure introduced by Chiarelli et al. (2016) was used for the inverse model. This allowed light intensity measurements to be localized in voxel space.

PReFx was computed as the area under the pulse wave between the peak systole and peak diastole, normalized for interbeat interval and peak amplitude to avoid confounds, and then subtracted of .5 (a value that would correspond to a hypothetical “linear” relaxation function, indicating constant speed of relaxation of the arterial wall during the interval between peak systole and diastole). As mentioned, PReFx is more positive for elastic arteries, and less positive (or even negative) for stiffer arteries. PReFx was estimated for each voxel for which the sensitivity (measured by the average Jacobian) was greater than 1/1,000 (60 dB) of the maximum value, allowing us to disregard voxels too deep to provide useful data (approximately >3 cm from the scalp) as well as voxels that were not covered by the optical montage. In addition, only voxels within the cortex (as identified by FreeSurfer) were considered. PReFx was computed as the average value across the 50 regions of the Desikan-Killiany atlas covered by the optical montages. Thus, we employed a global measure of PReFx to quantify cerebral arterial elasticity in this study.

##### Functional connectivity.

The resting-state MRI scans underwent standard fMRI preprocessing and FC analyses based on recommendations by Power et al. (2014) to reduce motion artifacts. The preprocessing steps were carried out using SPM12 (http://www.fil.ion.ucl.ac.uk/spm). Specifically, the T2*-weighted images for each run were slice-time corrected, realigned, and coregistered to each subjects’ structural MRI. The scans were then normalized to the MNI152 template brain by using 3-mm resampling. FC processing steps were then carried out in MATLAB. A censoring mask was first created by marking high motion frames using a variant of frame-wise displacement (*FD*), referred to here as *FDfilt*. *FDfilt* was calculated by low-pass filtering the six motion parameters at 0.1 Hz, finding the frame-wise displacement for each of them and then summing the absolute values across them for each frame. We chose to use *FDfilt* as it is more sensitive to differentiating motion from respiration as compared with *FD* (Fair et al., 2018). Any frames with *FDfilt* > .1 were marked as high motion. The first and last five frames each run were also marked for removal, as were any contiguous segments less than five frames long. Next, each run was demeaned, followed by nuisance regression ignoring the frames in the censoring mask. Nuisance variables included the global signal, average cerebral spinal fluid, and average white matter time courses, and the Volterra expansion of the six motion parameters (Friston, Williams, Howard, Frackowiak, & Turner, 1996; i.e., the 6 motion parameters of current and preceding volumes, and each of these values squared). After nuisance regression, data were linearly interpolated across censored frames to preserve data integrity for the next step of band-pass filtering, where a Butterworth filter (band-pass: 0.009–0.08 Hz) of order 1 was applied. The functional images were then spatially smoothed using a 6-mm FWHM Gaussian kernel. Finally, the frames initially marked for censoring that were temporarily interpolated with data were removed. To ensure that we had sufficient data for each participant, only participants with at least 50 frames remaining for each of the two runs were used (>100 frames total). As a result, two participants were excluded. The average number of frames remaining for our final sample of participants (*N* = 46 after the various post–data collection exclusions; see section *Participants*) across both runs was 297.91 frames (range = 169–320; see section S1.1 in the Supporting Information for how much censoring was done using solely the FDfilt exclusion criterion; C. Gratton et al., 2018, 2019).

We based our regions of interest (ROIs) on previous work, which sorted 264 brain areas into functionally distinct networks (Power et al., 2011). We focused on 11 out of the original 14 networks. Specifically, we used the 219 ROIs associated with the auditory, cingulo-opercular, dorsal attention, default, fronto-parietal, memory-related, sensorimotor hand, sensorimotor mouth, salience, ventral attention, and visual networks, leaving out the 45 ROIs that were undefined or belonging to the cerebellar and subcortical networks. The reason why subcortical networks were excluded from the analysis is because these networks cannot be easily explored with pulse-DOT measures (which tend to be limited to cortical areas).

The average time series within a 10-mm-diameter sphere was extracted for each of these 219 ROIs for each subject. A Fisher*Z*-transformed matrix was created for each subject based on the correlations between the time series for each pair of ROIs. To avoid using negative correlations, since their biological significance is currently debated (e.g., Murphy, Birn, Handwerker, Jones, & Bandettini, 2009), and to replicate the methods used by Chan et al. (2014) in their study of age-related differences in network segregation, all negative values in each participants’ *Z* -matrix were set to zero. All further FC analyses were derived from these Fisher *Z* -transformed matrices.

System segregation was calculated using the formula of Chan et al. (2014) (i.e., system segregation $=\frac{{\bar{Z}}_{w}-{\bar{Z}}_{b}}{{\bar{Z}}_{w}}$, where ${\bar{Z}}_{w}$ is the mean of the within-system Fisher *Z*-transformed *r’s*, and ${\bar{Z}}_{b}$ is the mean of the between-system Fisher *Z*-transformed *r’s*). Segregation was measured separately for association systems (i.e., cingulo-opercular, dorsal attention, default, fronto-parietal, memory-related, salience, and ventral attention networks) and sensorimotor systems (i.e., auditory, sensorimotor hand, sensorimotor mouth, and visual networks).

Finally, it should be noted that the above seeds from Power et al. (2011) were derived using younger adult samples, as is true of most other available parcellations. C. Gratton et al. (2018) have shown that this network assignment of nodes holds well even in older adults and people with Parkinson’s disease. Han and colleagues (2018) recently established age cohort–specific parcellations to further improve the validity of rsFC across cohorts. Thus, we also calculated association and sensorimotor system segregation by using the parcellations of Han et al. (2018). Method and results obtained using the parcellations of Han et al. (2018) are presented as Supporting Information (see sections S2.1 and S2.2 and related tables and figures). In brief, similar patterns of results were obtained across the two parcellation approaches.

### Statistical Analyses

Based on our hypotheses, we first performed pairwise correlations between age and all other factors in the model to ensure that we could replicate the previously demonstrated effects of aging. Specifically, we computed pairwise correlations between age and PReFx, WMSAs, cortical thickness, association system segregation, sensorimotor system segregation, and each of the a priori–defined cognitive construct scores.

Next, we measured the relationship between segregation and the other brain factors (i.e., cerebral arterial elasticity, WMSAs, cortical thickness). Specifically, we performed separate pairwise comparisons between the two segregation indices (association system and sensorimotor system segregation) and measures of WMSAs, cortical thickness, and PReFx.

Finally, we investigated the relationship between rsFC and cognition by correlating the six cognitive constructs defined a priori separately for association system segregation and sensorimotor system segregation.

For all of the above analyses, we report one-tailed *p* values since we had specific hypotheses about the direction of the age effects. Furthermore, we controlled for motion for both measures of system segregation (i.e., association or sensorimotor) by partialing out mean *FDfilt* as a control variable.

We also computed correlations of all the relevant variables in the study with sex. All these correlations were very small (|*r′s*| < .25), and none of them was significant at *p* < .05 level (with the exception of intracranial volume, which was used in the computation of the WMSA score; the correlation of the adjusted WMSA score with sex was, however, not significant; *r* = −.187, *p* = .212). For these reasons we decided not to use sex as a covariate in the current study, in order not to reduce the degrees of freedom of the analyses unnecessarily.

## Results

The correlation matrix including all variables measured in the study is presented in Table 2. Table 3 reports the same matrix of correlations with age partialed out.

**Table 2.** Complete correlation matrix

	Age	PReFx	WMSAs	Cortical thickness	Association system segregation	Sensorimotor system segregation	Episodic memory	Processing speed	Verbal fluency	Working memory	Reasoning
PReFx	−0.381**										
WMSAs	0.427**	−0.314*									
Cortical thickness	−0.629**	0.154	−0.326*								
Association system segregation	−0.707**	0.525**	−0.378**	0.563**							
Sensorimotor system segregation	−0.425**	0.211	−0.383**	0.346*	0.504**						
Episodic memory	−0.454**	0.234	−0.168	0.392**	0.450**	0.251					
Processing speed	−0.091	−0.048	−0.295*	0.181	0.184	0.322*	0.334*				
Verbal fluency	0.131	0.140	0.097	−0.070	0.047	0.209	0.008	0.249			
Working memory	−0.223	0.045	−0.263	0.269	0.196	0.442**	0.345*	0.565**	0.248		
Reasoning	−0.601**	0.092	−0.313*	0.437**	0.529**	0.404**	0.431**	0.294*	−0.085	0.498**	
Verbal ability	0.415**	−0.182	0.283	−0.157	−0.280	−0.087	0.070	−0.140	0.162	0.187	0.061

*Note.* PReFx = pulse relaxation function; WMSAs = white matter signal abnormalities.

*p < .05; **p, both one-tailed.

**Table 3.** Complete correlation matrix after partialing out age

	PReFx	WMSAs	Cortical thickness	Association system segregation	Sensori-motor system segregation	Episodic memory	Perceptual speed	Verbal fluency	Working memory	Reasoning
WMSAs	−0.180									
Cortical thickness	−0.120	−0.081								
Association system segregation	0.340*	−0.176	0.328*							
Sensorimotor system segregation	0.066	−0.269	0.165	0.370*					
Episodic memory	0.074	0.032	0.153	0.150	0.064					
Perceptual speed	−0.090	−0.285	0.160	0.216	0.337	0.330				
Verbal fluency	0.207	0.046	0.016	0.064	0.246	0.076	0.264			
Working memory	−0.044	−0.191	0.170	0.116	0.411*	0.281	0.561*	0.287		
Reasoning	−0.185	−0.078	0.095	0.232	0.229	0.222	0.301	−0.007	0.467*	
Verbal ability	−0.028	0.129	0.147	0.001	0.099	0.318	−0.113	0.119	0.315	0.428*

*Note.* PReFx = PReFx = pulse relaxation function; WMSAs = white matter signal abnormalities.

*p < .05.

### Effects of Aging

**Figure 1.**
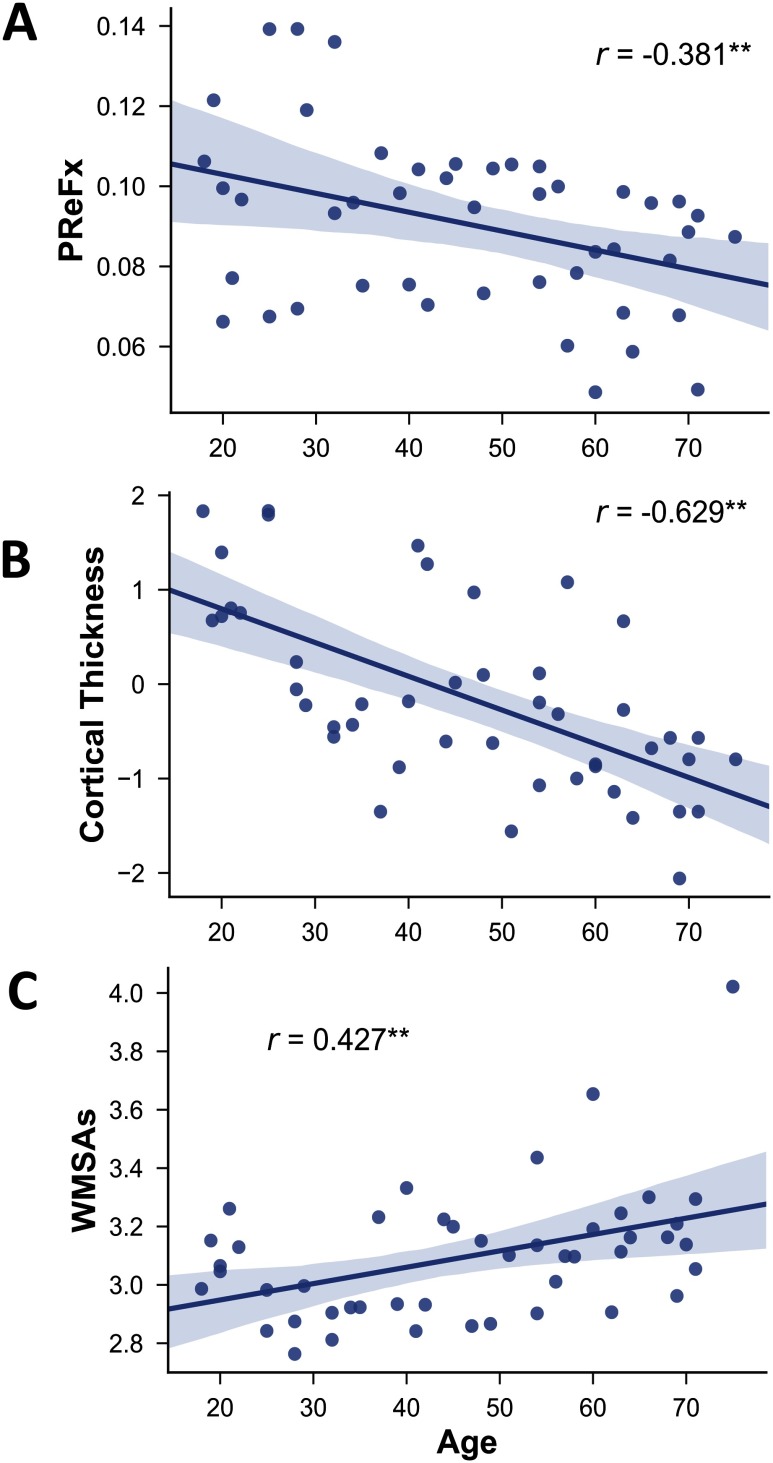
Relationship between age and PReFx (A), cortical thickness (B), and WMSAs (C). Shading indicates the 95% bootstrap confidence interval for the linear regression function (solid line); **p < .01. PReFx = pulse relaxation function; WMSAs = White matter signal abnormalities.

**Figure 2.**
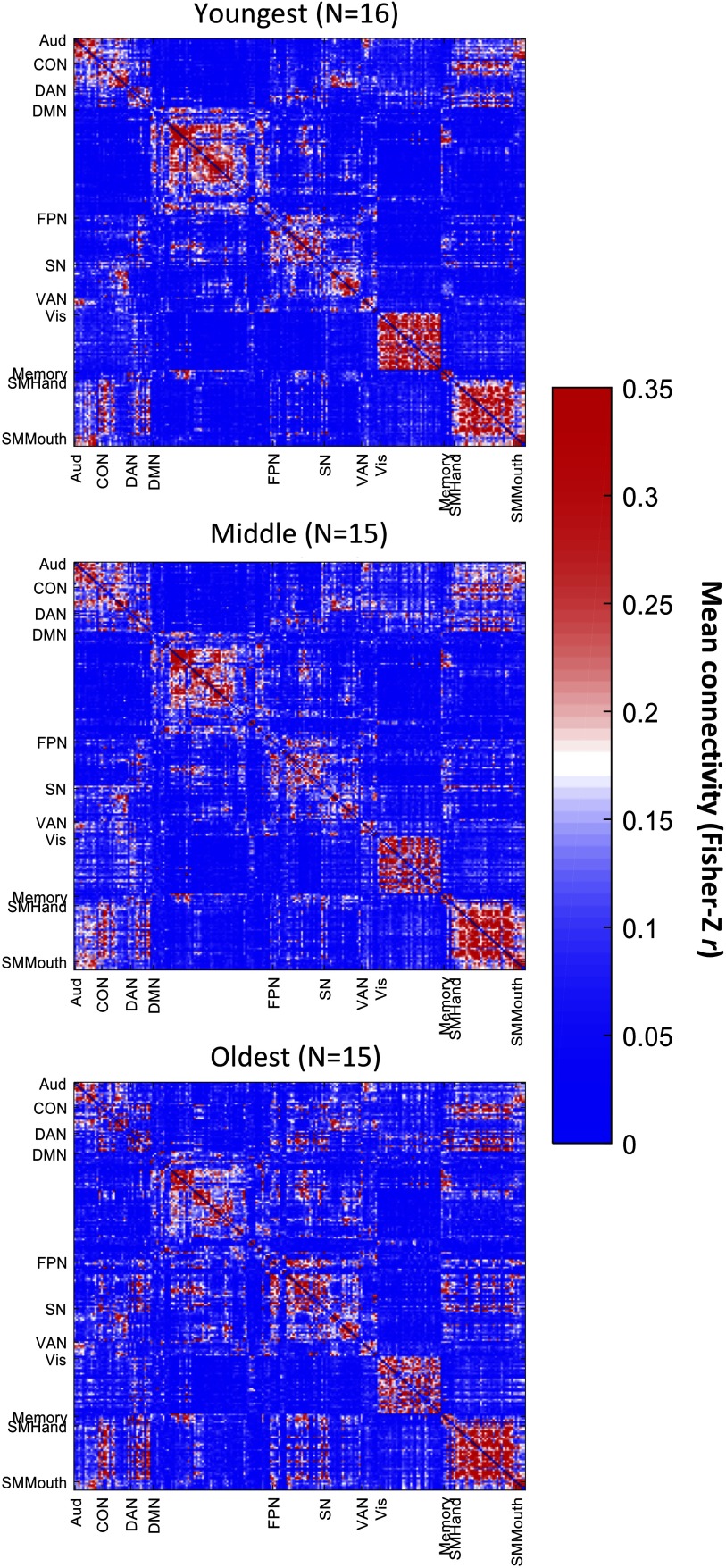
Mean functional connectivity matrices for younger (age range = 18–37 years, *N* = 16), middle-aged (age range = 39–57 years, *N* = 15), and older adults (age range = 58–75 years, *N* = 15). Color scale indicates the mean Fisher *Z*-transformed connectivity values. Aud = auditory network; CON = cingulo-opercular network; DAN = dorsal attention network; DMN = default mode network; FPN = fronto-parietal network; SN = salience network; VAN = ventral attention network; Vis = visual network; Memory = memory network; SMHand = sensorimotor hand network; SMMouth = sensorimotor mouth network.

**Figure 3.**
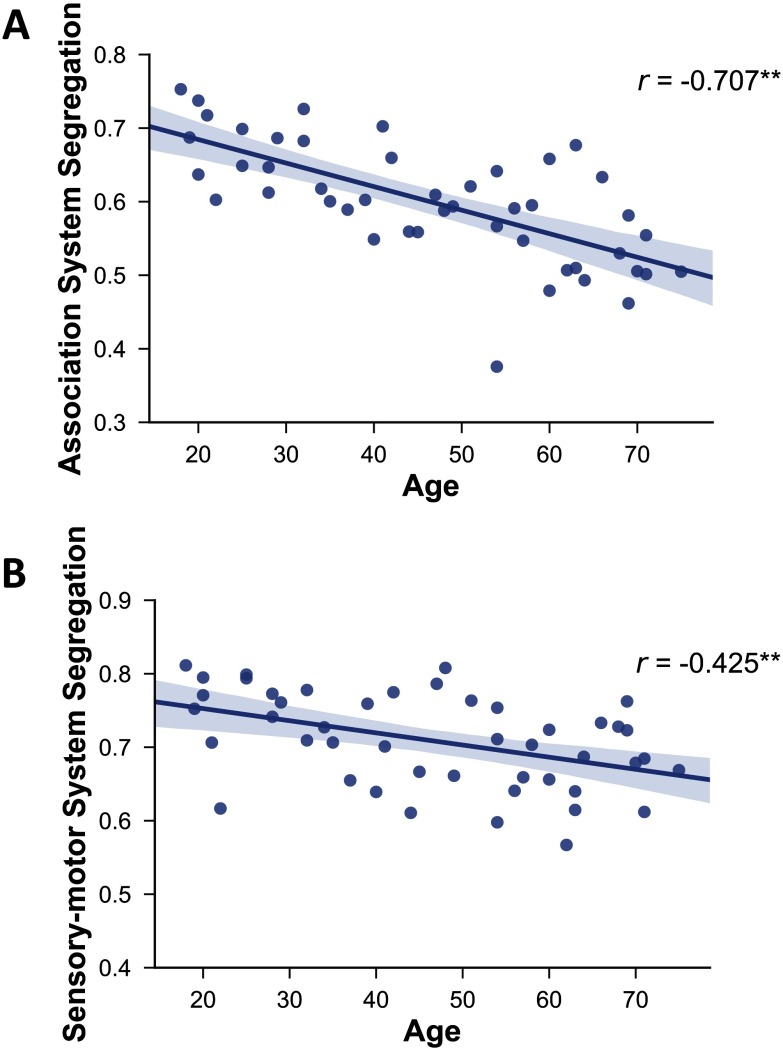
Relationship between age and association system segregation (A) and sensorimotor system segregation (B). Shading indicates the 95% bootstrap confidence interval for the linear regression function (solid line); **p < .01.

As expected, older age was associated with decreased PReFx and cortical thickness (*r*(44) = −.381, *p* = .005; Figure 1A; and *r*(44) = −0.629, *p* < .0001; Figure 1B), and with increased WMSAs (*r*(44) = .427, *p* = .002; Figure 1C). Furthermore, aging was related to reduced segregation (see Figure 2 and Figure 3), especially for association networks, as previously reported by Chan et al. (2014). Specifically, there was an age-related decrease for association system segregation (*r*(43) = −.707, *p* < .0001) and sensorimotor system segregation (*r*(43) = −.425, *p* = .002). However, the decrease was larger for association networks than for sensorimotor networks, as confirmed by a paired two-sample *t*-test (*t*(44) = −10.510, *p* < .001).

The relationship between cognition and aging varied depending on the cognitive construct. Whereas performance for episodic memory and reasoning decreased with age (*r*(44) = −0.454, *p* = .001 and *r*(44) = −0.601, *p* < .0001, respectively), verbal ability increased with age (*r*(44) = 0.415, *p* = 0.003), as is typically found (Baltes, Lindenberger, Schubert, Stober, & Weilandt, 1997). There were no significant age effects for the other cognitive constructs after correcting for multiple comparisons.

### Relationship Between PReFx, WMSAs, Cortical Thickness, and System Segregation

**Figure 4.**
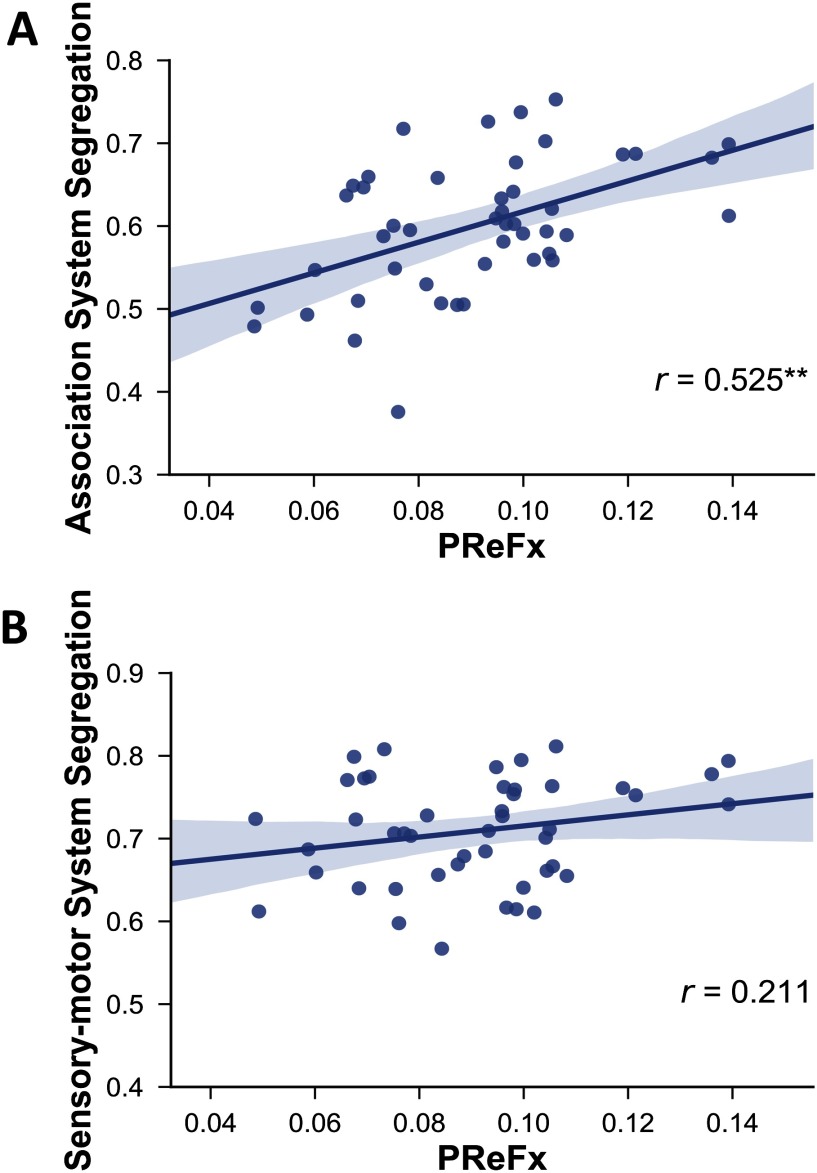
Relationship between PReFx and association system segregation (A) and sensorimotor system segregation (B). Shading indicates the 95% bootstrap confidence interval for the linear regression function (solid line); **p < .01. PReFx, pulse relaxation function.

Our results revealed that, as predicted, greater network segregation was related to greater arterial elasticity (Figure 4). Specifically, PReFx was significantly correlated with association system segregation (*r*(43) = .525, *p* < .001). The correlation between PReFx and sensorimotor system segregation was in the expected direction but did not reach statistical significance (*r*(43) = .211, *p* = .087).

**Figure 5.**
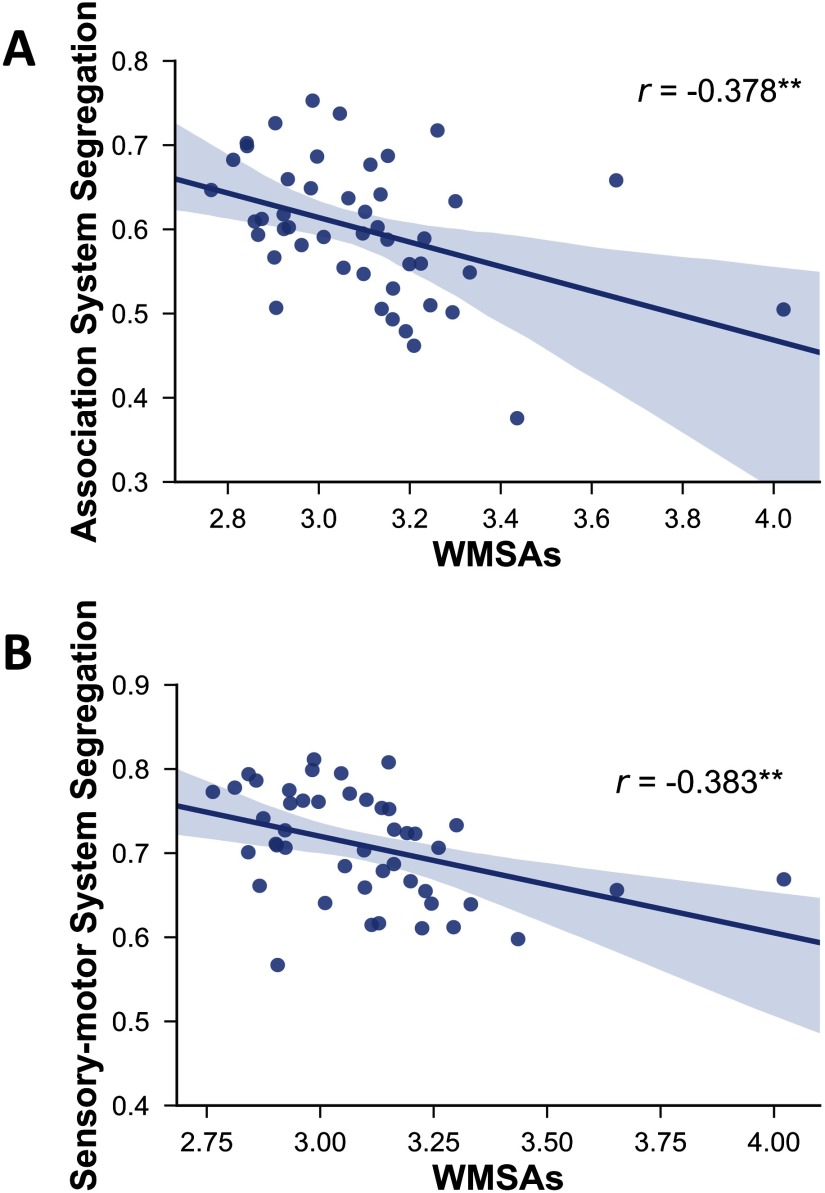
Relationship between white matter signal abnormalities (WMSAs) and association system segregation (A) and sensorimotor system segregation (B). Shading indicates the 95% bootstrap confidence interval for the linear regression function (solid line); **p < .01. Note that these relationships remain significant (*r* = −0.477 and *r* = 0.414, respectively, *p* < .01 for both) when the two extreme values are excluded.

Furthermore, and also as predicted, our results showed that network segregation was significantly associated with WMSAs (Figure 5). Specifically, WMSAs were negatively correlated with association system segregation (*r*(43) = −.378, *p* = .006) and sensorimotor system segregation (*r*(43) = −.383, *p* = .006).

**Figure 6.**
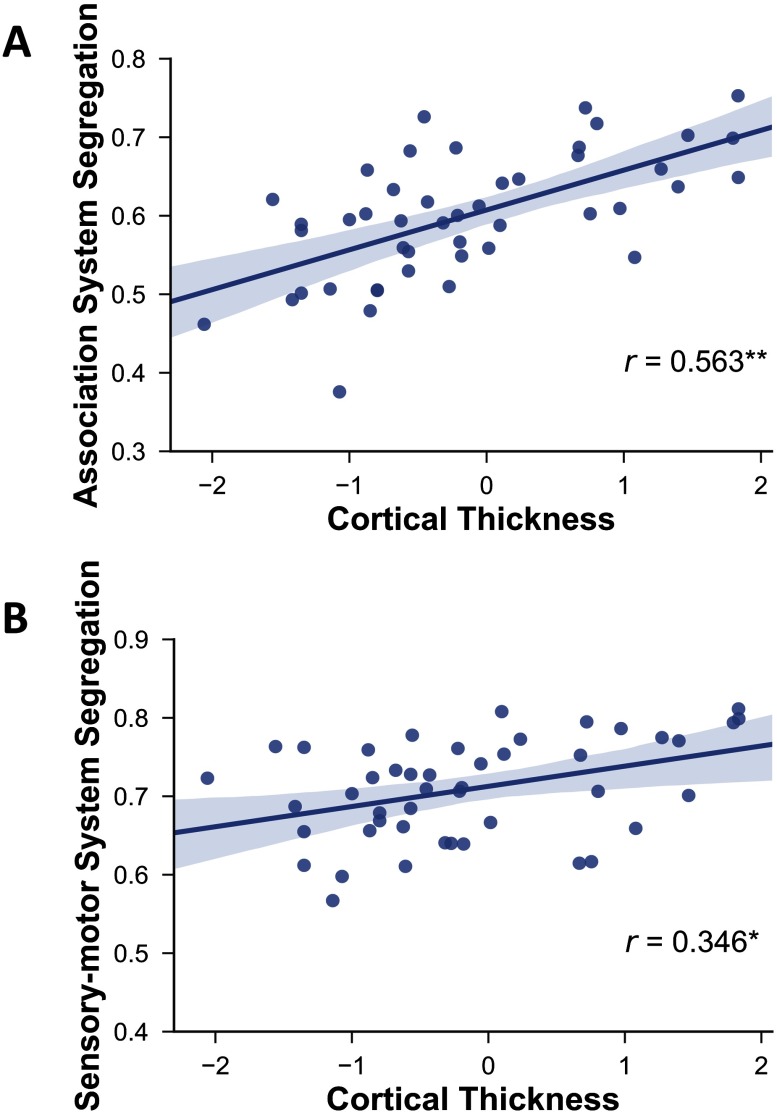
Relationship between cortical thickness and association system segregation (A) and sensorimotor system segregation (B). Shading indicates the 95% bootstrap confidence interval for the linear regression function (solid line); *p < .05, **p < .01.

**Figure 7.**
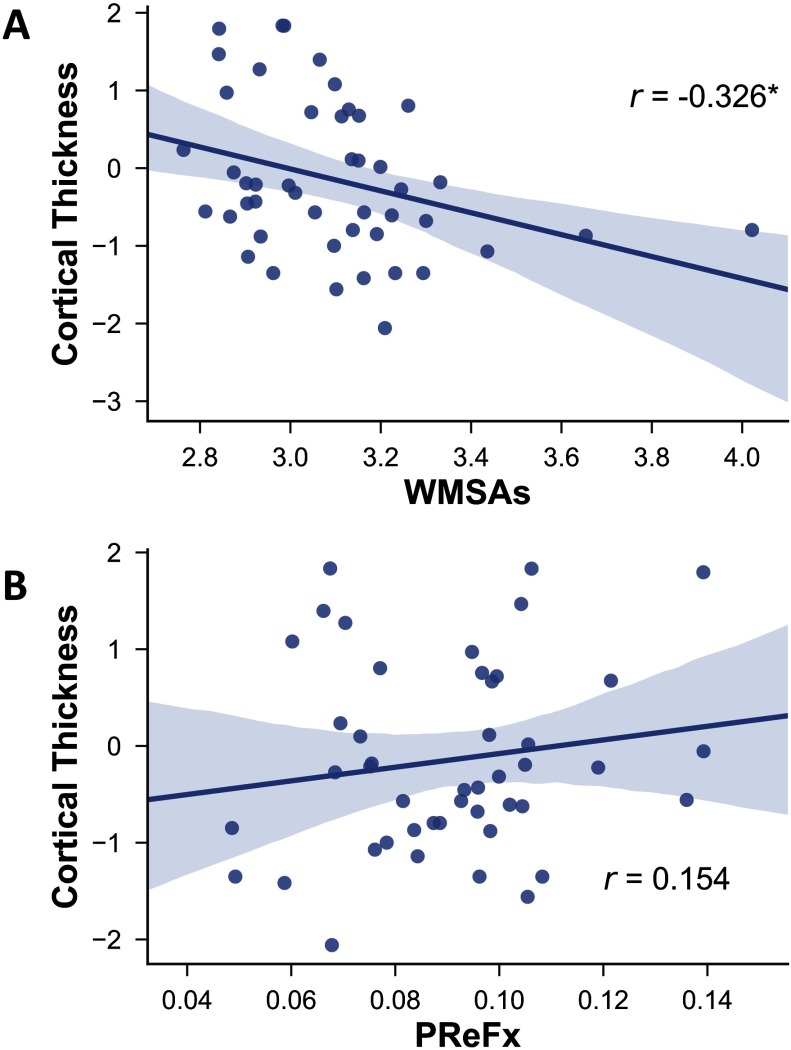
Relationship between cortical thickness and WMSAs (A) and PReFx (B). Shading indicates the 95% bootstrap confidence interval for the linear regression function (solid line); *p < .05. PReFx = pulse relaxation function; WMSAs = white matter signal abnormalities.

Finally, network segregation was also positively associated with cortical thickness (Figure 6; cortical thickness vs. association system segregation: *r*(43) = .563, *p* < .0001; vs. sensorimotor segregation: *r*(43) = .346, *p* = .012). However, although greater cortical thickness was associated with fewer WMSAs as expected (*r*(43) = −0.326, *p* = 0.016; Figure 7), it was not significantly correlated with PReFx (*r*(43) = .154, *p* = .162; Figure 7B).

Table 3 reports the correlation matrix across all the variables in the study after partialing out age. Notably, the correlations of association system segregation with PReFx and cortical thickness remain significant after removing the effect of age. Furthermore, the relationships between sensorimotor system segregation and working memory also remain significant. Finally, several of the correlations between cognitive constructs remain significant even after partialing out age.

### Relationship Between Network Segregation and Cognition

Similar to the correlations with age, episodic memory and reasoning were the only cognitive constructs significantly correlated with rsFC. Specifically, higher episodic memory performance was correlated with greater association system segregation (*r*(43) = .450, *p* = .001), whereas higher reasoning performance was correlated with both greater association system segregation (*r*(43) = .529, *p* < .001) and greater sensorimotor segregation (*r*(43) = .404, *p* = .004). Higher sensorimotor system segregation was also correlated with better working memory (*r*(43) = .442, *p* = .002). However, there was no relationship between verbal ability and segregation measures. Network segregation was related to other cognitive constructs, but these correlations were no longer significant after correcting for multiple comparisons by adjusting for the number of cognitive constructs (see Table 2). Specifically, by using a Bonferroni approach, the alpha-rejection criterion (*p* = .05) was divided by the number of cognitive constructs (6), so the adjusted alpha-rejection levels for significant cognitive-related results is *p* = 0.0083.

### Summary and Exploratory Integration of Results

**Figure 8.**
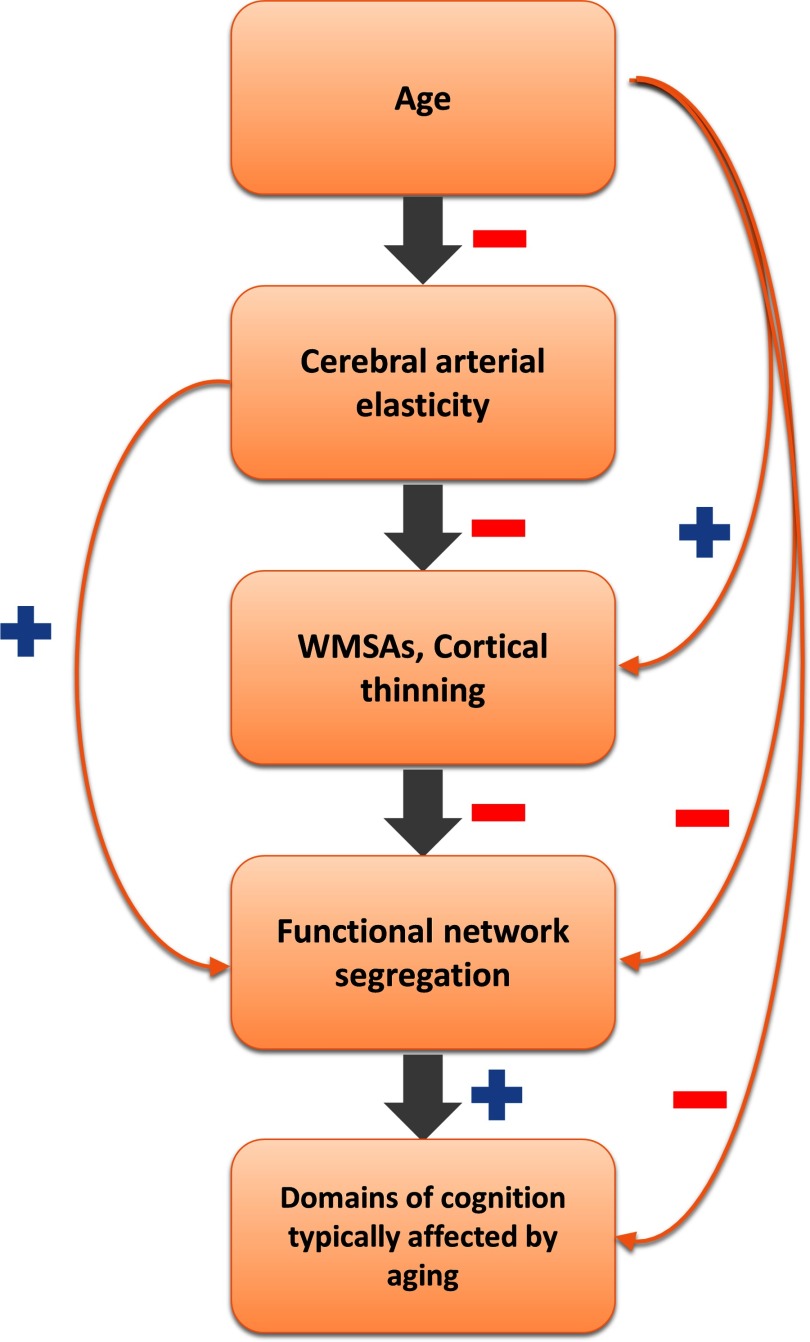
Schematic representation of an exploratory hierarchical cascade of effects. The sign next to each arrow indicates the direction of the relationship between pairwise variables (red minus sign = negative relationship; blue plus sign = positive relationship). The main hypothesized cascade of effects is indicated by the gray arrows. Pairwise relationships between the levels are indicated by the orange arrows. WMSAs = white matter signal abnormalities.

The results presented thus far are consistent with the presence of a cascade of phenomena, hierarchically linking the variables under study (Figure 8). This view (in line with others present in the literature, e.g., Barnes, 2015; de la Torre, 2012) proposes that aging is linked to declines in cerebral arterial elasticity (as measured here by *decreases* in PReFx), which, in turn, are tied to deterioration of white (as indexed by *increased* WMSAs) and gray matter structures (measured as *reduced* cortical thickness), due to reduced perfusion, decreases in neurotrophic factors (such as brain derived neurotrophic factor, BDNF, and vascular endothelial growth factor, VEGF; Voss et al., 2013a; Voss, Vivar, Kramer, & Van Praag, 2013b), and neuronal and myelin loss. These factors consequently weaken brain function and network organization, as measured by the *reduced* rsFC network segregation, and finally manifest in lower cognitive performance, especially in those cognitive domains that are most vulnerable to aging (e.g., episodic memory and reasoning/fluid intelligence).

Note that this hypothesized cascade does not exclude that aging might influence structural brain integrity through other pathogenetic mechanisms, such as the development of plaques and neurofibrillary tangles, neuroinflammation, and so on. It is also important to note that each of the levels in the cascade (cerebrovascular, anatomical, functional, and cognitive) is likely multidimensional, and that there are multiple ways in which each level can be quantified. For example, functional network organization may be quantified using segregation (Chan et al., 2014), participation coefficient, and modularity (Geerligs et al., 2015; see Sporns & Betzel, 2016, for an overview). However, our aim here is to highlight some specific links between pairs of consecutive elements along the chain depicted in Figure 8, rather than to provide an exhaustive coverage of all possible routes to cognitive aging.

In this paper, we have provided substantial evidence for *correlational* links between any two consecutive levels within the chain depicted in Figure 8. However, the cross-sectional nature of the study and limited sample size preclude the use of a systematic multilevel mediation analysis. Instead, here we use an indirect approach to provide a preliminary exploration of the hypothesized cascade. In fact, this hypothetical chain also suggests that the relationships between adjacent levels in the chain should be overall stronger than those between nonadjacent levels, with the smallest correlations for the levels that are furthest apart. Thus, an examination of the patterns of correlations across adjacent versus nonadjacent levels might provide some very preliminary evidence in support of the proposed hierarchy.

An exception to this expected correlation pattern might occur for the age variable. This is due to two reasons: (a) age can influence all other levels in multiple ways, and not necessarily only through the chain proposed in Figure 8 (e.g., age could influence cognition via life-long learning and other cohort effects); and (b) age can be measured with almost errorless precision, which is not the case for any of the variables representing other levels, engendering de facto higher correlations because of its lack of error variance. In other words, were we to include age in this final exploratory test, age would dominate (and therefore likely obscure) all other relationships. As such, we omitted age when examining the patterns of correlations across levels (see Table 3 for effect of partialing out age on relationships between the other variables in the study).

Note that multiple measures are available for several levels. For example, for the structure level, we combined the correlations of each of the other levels with cortical thinning and WMSAs (whose sign was changed to maintain coherence with other variables). Similarly, for the functional segregation level, we combined correlations involving associative and sensorimotor networks. Finally, for the cognitive level we combined the correlations for episodic memory and reasoning. To combine the variables for each level, we did the following: (a) The values for each variable within each level were standardized; (b) if needed, their sign was changed to maintain coherence; (c) the standardized values were averaged together; and (d) correlations were computed across levels.

**Figure 9.**
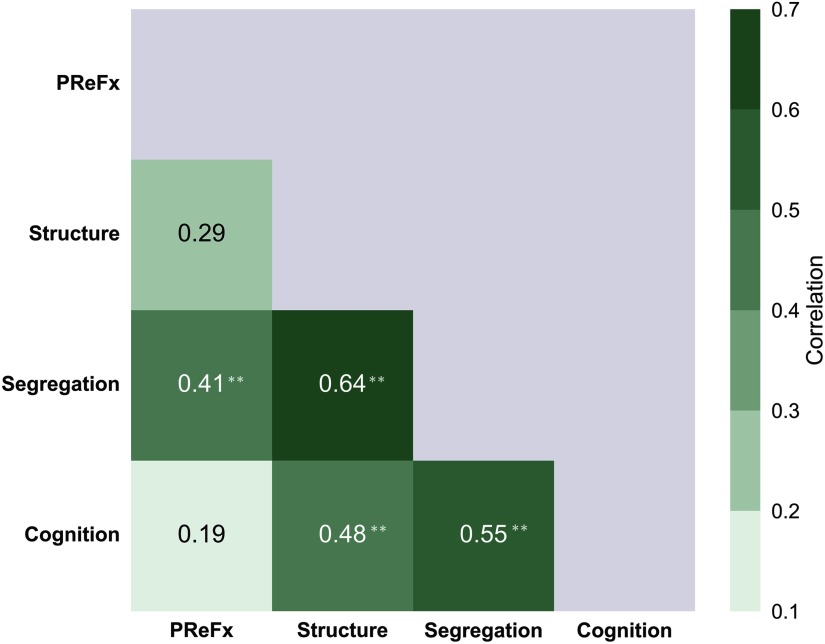
Absolute values for the integrated pairwise correlations between levels in the exploratory hierarchical model presented in Figure 8. The green shading indicates the strength of the correlations, based on the scale presented on the right; **p < .01. PReFx = pulse relaxation function.

The resulting table of correlations is presented in Figure 9. As predicted, correlations tended to be higher (average *r* = .51) between adjacent levels (1-off from the main diagonal in Figure 9), intermediate between levels separated by one level (average *r* = .45), and smallest between levels separated by two levels (*r* = .19). This apparent pattern is consistent with the predictions of the hierarchical model. To provide quantitative support for this qualitative impression, we performed a bootstrap analysis in which we generated 10,000 samples of *N* = 46, taken from the subject pool with replacement. For each bootstrap sample, we calculated the same 6-value correlation matrix presented in Figure 9, and then calculated the mean (Fisher-transformed) correlations of the elements 1-, 2-, and 3-off the diagonal, defined as above. Finally, we compared the differences between the actual values (from Figure 9) with the distribution of the corresponding differences obtained with the bootstrap approach. The results indicated that both the 1-off and the 2-off correlation values were significantly greater than the 3-off (*p* = .0021 and *p* = .0170, respectively). However, the difference between the 1-off and the 2-off values was not significant (*p* = .2109).

## Discussion

This study examined the interrelationships between cerebrovascular, structural, and functional factors associated with age-related differences in cognition. Specifically, we found that cerebrovascular elasticity (as measured by pulse-DOT PReFx) and structural integrity (as measured by WMSAs and cortical thickness) were related to functional network segregation (taken as an indicator of network integrity) and to age-related differences in cognitive domains that are typically affected by aging (episodic memory and reasoning/fluid intelligence). By using pairwise comparisons, we showed that aging is related to reduced cerebral arterial elasticity, increases in WMSAs, reduced cortical thickness, and reduced network segregation (especially in association systems, compared with sensorimotor systems). In turn, these differences are related to reduced cognitive performance in the aforementioned domains. Finally, to integrate these correlational results and put them in context, we used an exploratory hierarchical model as a reference framework, and showed that adjacent levels within the model were more strongly correlated than distant ones, when the relationship with age was not considered. Further research, using larger and/or longitudinal samples, will be needed to allow for a more formal testing of this model.

A novel and central aspect of the current study is that we showed, for the first time, a robust relationship between optical measures of the elasticity of cerebral arteries and associative network segregation, extending the findings of Chan et al. (2014). This relationship remained significant even after controlling for the effects of age (Table 3), supporting the idea that individual differences in cerebrovascular health may explain important variations in functional brain organization, irrespective of age.

As mentioned, the relationships between aging, network segregation, and cognitive performance reported in this paper closely replicated results previously reported by Chan et al. (2014) in a larger sample, demonstrating the robustness and replicability of these findings across different scanner sites and different samples of participants. Importantly, we also replicated their finding of a significant relationship between episodic memory and association system segregation, despite using a subset of different tests for these constructs, demonstrating that the relationships between system segregation and cognition may be generalizable even across nonidentical cognitive tasks. In addition, we found that system segregation (both association and sensorimotor) was also correlated with reasoning.

The cognitive tests used in this study focused on working memory and reasoning, consistent with the extensive literature, indicating that these domains of cognition are more affected by aging than others. Specifically, fluid intelligence (of which reasoning is a key component) is more vulnerable to aging compared with crystallized intelligence (which includes vocabulary and general knowledge). The latter has been shown to be relatively unaffected by aging until very late in life, and we did not expect any age-related differences, especially given the maximum 75 years of age of our sample. Thus, within this context, our results are typical: they indicate that domains of cognition that are especially affected by aging (i.e., episodic memory and reasoning) are also affected by cerebrovascular health.

Because of the relatively small sample used in this study, we primarily relied on pairwise correlations between variables. Although our results are consistent with the hierarchical model presented in Figure 8, we could not explicitly test it, because of insufficient power for implementing mediation analyses. In addition, the cross-sectional nature of the current study further limits inferences regarding potential causal links. Future research should investigate this model by using a larger longitudinal sample, paired with mediation analyses, to fully explore the proposed relationships.

It is also important to note that the exploratory model presented in Figure 8 is based on relationships between selected measures within each level/factor instead of testing for a number of possible measures that may be available for each level (e.g., functional network organization can be measured in several other ways). Although beyond the scope of this paper, it is possible that a more thorough investigation of other measures within each level would allow us to better refine the neurophysiological pathways that might be implicated. For example, age-related declines in cerebral perfusion may play a role in how a reduced arterial elasticity is tied to increases in WMSAs (Bernbaum et al., 2015; Brickman et al., 2011; Marstrand et al., 2002; van Dalen et al., 2016) and decreases in brain volume and memory performance (Alosco et al., 2013). Similarly, there is also evidence that the plasma level of VEGF is related to the incidence of WMSAs (Pikula et al., 2013), is predictive of baseline and longitudinal hippocampal volume and cognitive performance (Hohman & Jefferson, 2015), and may be modified by exercise training (Maass et al., 2016; Voss et al., 2013a). The current results are in line with this literature but do not fully explore these mechanisms.

Despite the above limitations, the reported findings are consistent with a hierarchical relationship between different cerebrovascular, structural, and functional variables. Demonstration of a hierarchical relationship between these variables may suggest that preservation of cognition in healthy aging could be tied to improvements on one or more of these levels. In other words, it is possible that the effects of interventions designed to delay the effects of age on cognition may influence this cascade, so that variations at any level may be reflected in differences not only at that level but also on subsequent levels in the hierarchy, including the ultimate outcome of improved (or at least delayed decline of) cognition. Indeed, it has been shown that physical exercise (which may modify arterial function) is linked to cortical thickness (Lee et al., 2016; Williams et al., 2017) as well as improved rsFC and cognition in older adults (Voss et al., 2010). In this sense, measures of intermediate levels within the proposed cascade may be used to demonstrate not only the efficacy of an intervention strategy but also point at some of the mechanisms involved in the process.

Along the same lines of reasoning, there is some evidence that rsFC measures may be useful for predicting intervention outcomes in older adults (Baniqued et al., 2018). It has been found that older adults exhibiting higher network modularity at baseline showed greater improvements in synthesizing complex information after cognitive training, and this effect was more pronounced for association systems compared with sensorimotor systems (Gallen et al., 2016). Furthermore, there is evidence that some interventions may promote changes in WMSAs, rsFC, and cognition as demonstrated by a study examining older adults after 6 months of resistance training, cognitive training, or both (Suo et al., 2016). Results demonstrated that resistance training improved scores on a clinical dementia scale, whereas cognitive training was linked to the stabilizing of memory performance. Interestingly, resistance training was linked to improvements (i.e., decreases) in WMSAs, although this was not linked to any improvements in behavior. Cognitive training, however, was linked not only to improved hippocampal rsFC but also to improvements in memory performance. Although untested, this finding may suggest that even though all these phenomena are affected by intervention, improvements in rsFC measures may manifest more clearly in cognitive performance than improvements in WMSA measures. Although this particular study employed a sample with mild cognitive impairment, it is still highly relevant to our understanding of these factors in normal aging. Together with converging evidence linking exercise to improvements in cerebrovascular function in older adults (see Voss, Nagamatsu, Liu-Ambrose, & Kramer, 2011, for a review), these studies suggest that multiple neurobiological factors may be improved by intervention, leading to preservation of cognitive function in older adults.

In conclusion, the current study investigates pairwise relationships between multiple neurophysiological factors that may explain some of the effects of aging on cognition. Specifically, we propose that aging compromises the cerebral vasculature, leading to declines in brain structure, which in turn reduce brain functional integrity and finally result in reduced cognitive performance (see also Barnes, 2015, and de la Torre, 2012, for similar models applied to Alzheimer’s disease). Although a more in-depth exploration of these relationships is needed, including explicit testing of causal links, the current findings suggest pathways by which cognition can be preserved through improvement of one or more of the factors.

## ACKNOWLEDGMENTS

We acknowledge the support of the Bioimaging Center of the Beckman Institute for Advanced Science and Technology at the University of Illinois at Urbana-Champaign (UIUC-BI-BIC). We are grateful to Dr. Gagan Wig and colleagues for providing access to their age-cohort-specific parcellations (Han et al., 2018).

## SUPPORTING INFORMATION

Supporting Information for this article is available at https://www.doi.org/10.1162/netn_a_00110.

## AUTHOR CONTRIBUTIONS

Tania S. Kong: Formal analysis; Investigation; Methodology; Software; Visualization; Writing - Original Draft. Caterina Gratton: Data curation; Methodology; Writing - Review & Editing. Kathy A Low: Data curation; Project administration; Supervision; Writing - Review & Editing. Chin Hong Tan: Data curation; Formal analysis; Writing - Review & Editing. Antonio M Chiarelli: Formal analysis; Methodology; Software; Writing - Review & Editing. Mark A Fletcher: Formal analysis; Writing - Review & Editing. Benjamin Zimmerman: Data curation; Writing - Review & Editing. Edward L Maclin: Data curation; Writing - Review & Editing. Bradley P. Sutton: Methodology. Gabriele Gratton: Conceptualization; Formal analysis; Funding acquisition; Methodology; Supervision; Writing - Review & Editing. Monica Fabiani: Conceptualization; Funding acquisition; Project administration; Supervision; Writing - Review & Editing.

## FUNDING INFORMATION

Monica Fabiani, National Institute on Aging, Award ID: R01AG059878. Gabriele Gratton, NIH, Award ID: R56MH097973. Gabriele Gratton, NCRR, Award ID: S10-RR029294.

## TECHNICAL TERMS

Diffuse optical tomography (DOT): DOT is an imaging method based on near-infrared light. Using a spectroscopic approach, it images changes in oxy- and deoxy hemoglobin. Data can be reconstructed in 3D (tomography).
Pulse-DOT: The arterial pulse wave measured with DOT. Three parameters of the pulse wave can be measured: amplitude, pulse transit time (analog to pulse wave velocity), and PreFx.
Pulse relaxation function (PReFx): A measure of the shape of the pulse wave, reflecting the relative overlap between the forward (systolic) and the backward (diastolic) waves. A small overlap, resulting in a large PreFx value, indicates high arterial elasticity.
Segregation: A measure used in graph theory for summarizing the relative independence of brain networks.
