# Supplementary material for: Age-related differences in functional brain network segregation are consistent with a cascade of cerebrovascular, structural, and cognitive effects
Source: Netw Neurosci. 2020 Feb 1;4(1):89–114. doi: 10.1162/netn_a_00110 (PMC7006874; doi:10.1162/netn_a_00110)
Supplement: Supplementary file 1 [file netn-04-89-s001.pdf]

Kong, T. S., Gratton, C., Low, K. A., Tan, C. H., Chiarelli, A. M., Fletcher, M. A., ... Fabiani, M. (2020). Supporting information for "Age-related differences in functional brain network segregation are consistent with a cascade of cerebrovascular, structural and cognitive effects." *Network Neuroscience*, *4*(1), 89-114.
https://doi.org/10.1162/netn_a_00110

SUPPLEMENTARY INFORMATION

AGE-RELATED CHANGES IN FUNCTIONAL BRAIN NETWORK SEGREGATION ARE ASSOCIATED TO A CASCADE OF CEREBROVASCULAR, STRUCTURAL AND COGNITIVE EFFECTS

Tania S. Kong[1,2]; Caterina Gratton[3,4]; Kathy A. Low[1]; Chin Hong Tan[1,5,6]; Antonio M. Chiarelli[1,7]; Mark A. Fletcher[1]; Benjamin Zimmerman[1]; Edward L. Maclin[1]; Bradley P. Sutton[1,8]; Gabriele Gratton[1,2]; Monica Fabiani[1,2]

[1]Beckman Institute, University of Illinois at Urbana-Champaign
[2]Psychology Department, University of Illinois at Urbana-Champaign
[3]Department of Psychology, Northwestern University
[4]Department of Neurology, Northwestern University
[5]Division of Psychology, Nanyang Technological University
[6]Department of Pharmacology, National University of Singapore
[7]Department of Neuroscience, Imaging and Clinical Sciences, University G. D'Annunzio of Chieti-Pescara
[8]Department of Bioengineering, University of Illinois at Urbana-Champaign

S1.1 Supporting information for the *Functional Connectivity* section

In the section on *Functional Connectivity* we reported the number of frames left after censoring, which were used in the final rsFC analyses. This final censoring considered not only the frames exceeding the FDfilt > .1 criterion, but also the removal of the first and last frames in each run and of sections containing less than 5 contiguous frames. In Table S4 we report the number of frames that were censored based solely on the FDfilt > .1 criterion.

Table S1. Descriptive statistics for the number of frames (out of the 180 frames in each run) deemed to have excessively high motion based on the FDfilt > .1 criterion.

	**Original participants (N = 49)**	**Final participants (N = 46)**
**Mean**	14.153	10.196
**Minimum number of frames excluded**	0	0
**Maximum number of frames excluded**	129	61
**Standard Deviation**	24.398	14.406

S2.1 Method for Han et al.'s (2018) parcellation

The results reported in the paper were based on Power et al. (2011)'s seeds, to match the methods used by Chan et al. (2014). A recent study by Han et al. (2018) established age-cohort-specific parcellations based on a sample with a wide age range. Therefore, we re-analyzed our data to determine the robustness of the findings across the two parcellation approaches.

To derive age-cohort-specific measures of segregation, we first assigned our participants to an age cohort based on their age (see table S1 for the age ranges and *Ns* for each cohort). Then, using their preprocessed data (see section 2.3.4), we derived an average time-series for each parcel in their age-cohort-specific parcellation. Each parcel was assigned to a network and network-type (association vs. sensorimotor) as determined by Han et al. (2018). Specifically, association systems included the cingulo-opercular, dorsal attention, default mode, frontoparietal, medial parietal, medial temporal parietal, superior temporal gyrus and ventral attention networks, while sensorimotor systems included the auditory, visual, hand somatosensory, and mouth somatosensory networks. A functional connectivity matrix was formed for each subject using their cohort-specific parcellations. Association and sensorimotor system segregation indices were calculated using Chan et al.'s (2014) formula (see section on *Functional Connectivity*).

Table S2 Labels and age ranges for each age cohort (based on Han et al.'s age-cohort-specific parcellations); YA = young adults, ME = middle-early, ML = middle-late, OE = old-early, OL = old-late); *N*= the number of participants in our sample assigned to each cohort-specific parcellation.

Han et al. (2018) cohort	Age range (years)	*N*
YA	20-34	14
ME	35-49	11
ML	50-64	13
OE	65-79	8
OL	80-93	0

S2.2 Results for the Han et al.'s (2018) parcellation

As illustrated by Figures S1a-d, the average functional connectivity matrices across participants for each age-cohort tended to exhibit decreased within-network FC (i.e., lower values for the diagonal same-network squares) and increased between-network FC (i.e., higher values for the off-diagonal between-networks rectangles) for older compared to younger age cohorts. Table S2 shows the correlations between each of the two segregation indices and the other variables in the model. These results suggest that the strength of the correlations between segregation and the other variables decreased slightly when using the age-cohort-specific parcellations compared to the Power et al.'s (2011) parcellations. In particular, the relationship between WMSAs and segregation was no longer significant. However, despite this decrease, the *pattern of correlations* between the segregation indices and the other factors in the model were

quite similar across the two parcellation approaches. To quantify this pattern similarity, we correlated the two *R* columns in Table S2, which were derived using the Han et al.'s (2018) parcellations, with the same columns derived using the Power et al. (2011) parcellation. The patterns of correlations between the factors in the model and the segregation indices derived from the two parcellations were very high (r = 0.971 for association system segregation; r = 0.964 for sensorimotor system segregation).

(a)

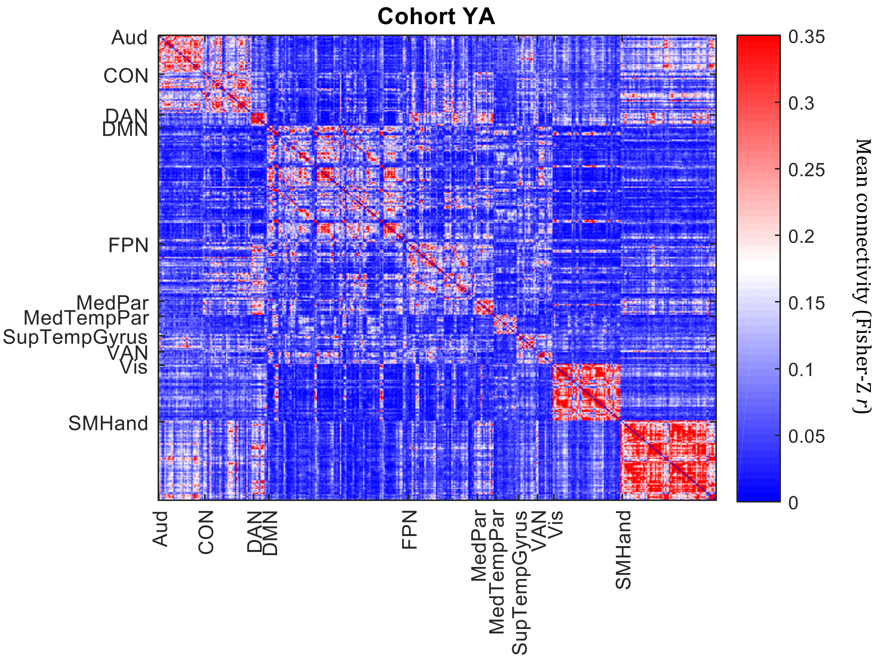

(b)

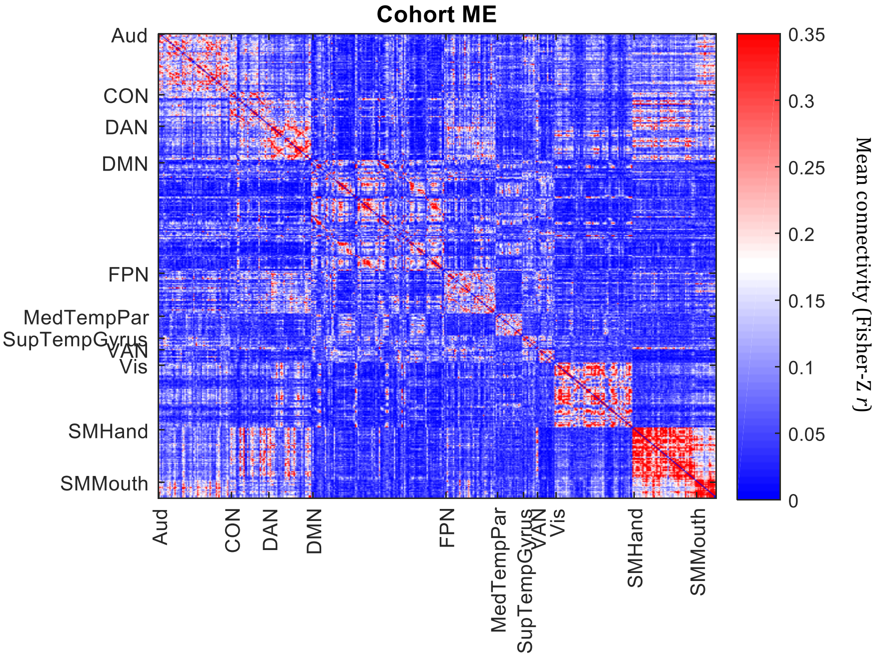

(c)

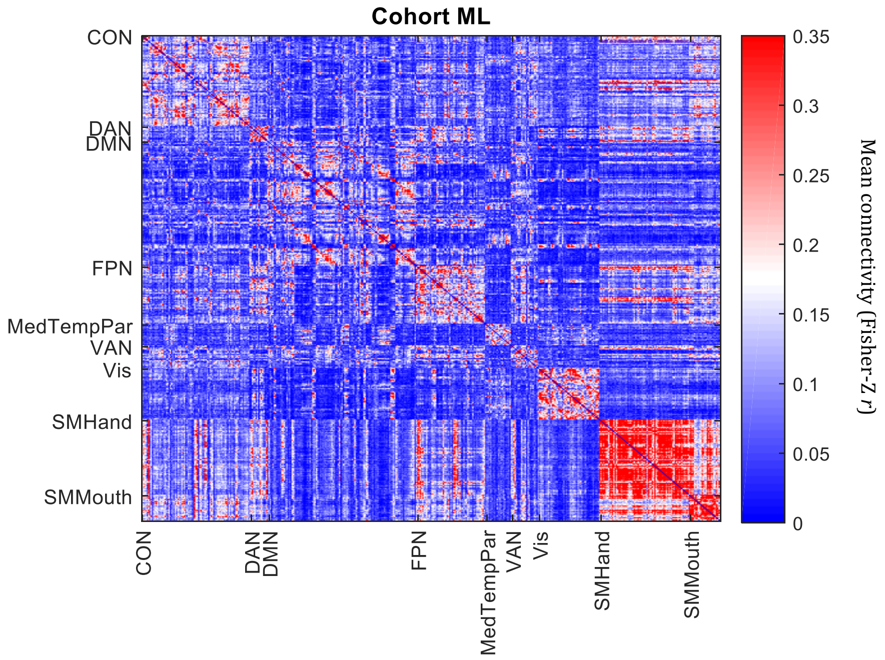

(d)

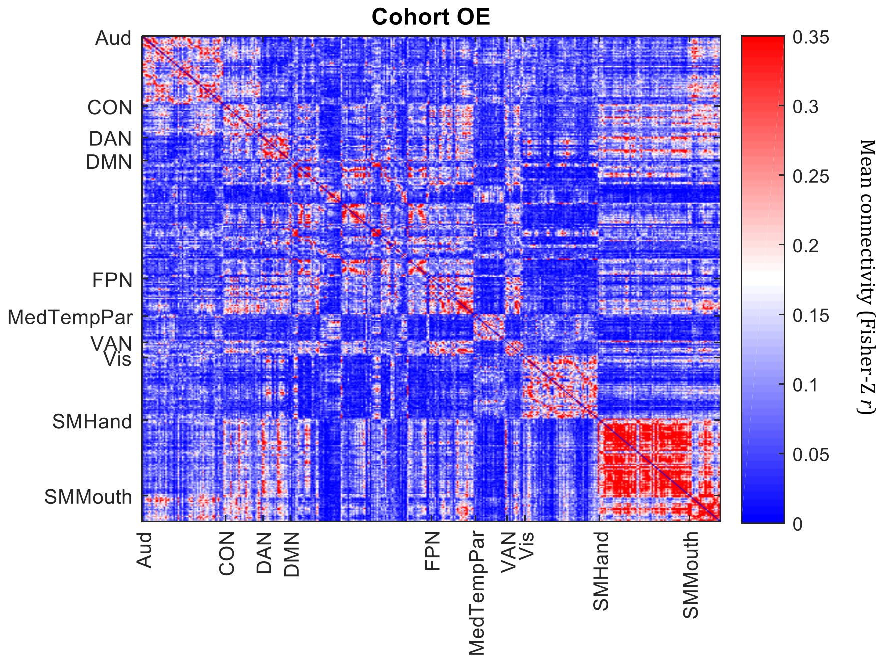

Figure S1. Average functional connectivity matrices for the (a) young adult cohort; (b) middle-early adult cohort; (b) middle-late adult cohort; and (c) old-early adult cohort.

Table S3 Pairwise correlations between association system and sensorimotor system segregation and the other variables in the model (with mean motion being controlled for). Based on the Han et al.'s parcellation approach.

	**Association System Segregation**		**Sensorimotor System Segregation**	
	R	***p***	***R***	***p***
**Age**	-0.657	0.000	-0.528	0.000
**PReFx**	0.357	0.016	0.227	0.133
**WMSA**	-0.179	0.238	-0.312	0.037
**Cortical thickness**	0.408	0.005	0.405	0.006
**Episodic Memory**	0.331	0.026	0.459	0.001
**Perceptual Speed**	0.142	0.352	0.406	0.006
**Verbal Fluency**	0.174	0.252	0.131	0.389
**Working Memory**	0.134	0.380	0.277	0.065
**Reasoning**	0.410	0.005	0.352	0.018
**Verbal Ability**	-0.138	0.364	-0.259	0.086
